# Being human as praxis: for people with learning disabilities

**DOI:** 10.1057/s41286-023-00159-6

**Published:** 2023-06-12

**Authors:** Dan Goodley

**Affiliations:** iHuman, School of Education, University of Sheffield, Sheffield, UK

**Keywords:** People with learning disabilities, Critical disability studies, Postcolonial studies, Sylvia Wynter, Human being, Praxis

## Abstract

The paper posits that *being human as praxis*—in relation to the lives of People with Learning Disabilities—offers a significant and original insight into critical and social theory across the social sciences and humanities. Drawing on postcolonial and critical disability theory I suggest that being human as praxis of People with Learning Disabilities is sophisticated and generative but is always enacted in a deeply disablist and ableist world. I explore being human as praxis in (i) a culture of disposability; (ii) the midst of absolute otherness and (iii) the confines of a neoliberal-ableist society. For each theme I start with a provocation, follow up with an exploration and end with a celebration (with the latter referencing the activism of people with learning disabilities). I conclude with some thoughts on simultaneously *decolonising* and *depathologising* knowledge production, the importance of recognition and writing *for* rather than *with* People with Learning Disabilities.

## Introduction

This paper sits with some contributions of postcolonial and critical disability scholarship that I believe are responsive to the lives and aspirations of People with Learning Disabilities.^1^ I draw upon a specific concept developed by the postcolonial scholar Sylvia Wynter (Wynter 1992, 2003, 2006; Wynter and McKittrick 2015): *being human as praxis* to read some aspects of the lives of People with Learning Disabilities.^2^ Following Wynter I approach the phenomenon of the human less as a noun and more as a verb^3^: human being. This dynamic, active and inclusive understanding of being human offers a significant and original insight for social theory across the social sciences and humanities. In turning to the human being as praxis of People with Learning Disabilities, this not only addresses intellectual ignorance (a consequence of ignoring people so-labelled in our social theory) but offers intellectual expansion (broadening our understandings of what it means to be human). I elaborate on human being as praxis in the lives of People with Learning Disabilities through reference to three themes (i) in a culture of disposability; (ii) in the midst of absolute otherness and (iii) in the confines of a neoliberal-ableist society. For each of these three themes I offer an analytically unique approach; I start with a provocation, follow up with an exploration and end with a celebration. In terms of the latter, I will draw on examples of activism from the self-advocacy movement in Britain: specifically four organisations whose work was absolutely crucial to People with Learning Disabilities especially during the recent pandemic. The paper concludes with some thoughts on simultaneously *decolonising* and *depathologising* knowledge production, the importance of recognition and the need for rigorous forms of co-production. I end with some reflections on writing *for* rather than *with* People with Learning Disabilities.

People with Learning Disabilities live deeply intersectional lives; cutting across gender, age, class, sexuality, race and place. Following Goodley et al. (2020, p. 3) I understand intersectionality ‘not simply in terms of an additive process of rolling together a myriad of oppressed positionalities (though such a move can be valuable) but as a liminal space where different forms of marginality and politicised responses work in tension, across and with a host of politicised offerings in very generative ways’. This paper will illuminate some of these generative possibilities. Interrogating the intersections of disability and race is essential. We know, for example, that People with Learning Disabilities in the UK from Black, Caribbean, African or Asian communities die at a younger age than those of a white ethnicity (White et al. 2022) and disabled People from LGBTQI + , Indigenous or Black communities have some of the worst health indices. There have been numerous examples of postcolonial disability theory that foregrounded the links between coloniality and disability (Grech 2015), unpacked cultural depictions of empowerment and inferiority (Jin 2022), interrogated racialised and disabled relationalities in the constitution of nation states (Soldatic 2015), enhanced disability justice in universities (Masitera 2020) and challenged hegemonic histories of institutionalisation and post-institutionalisation (Altermark 2017). My purpose in bringing together postcolonial and disability studies relates specifically to a desire to develop a deeper analytical understanding of dehumanisation and new conceptions of humanity in the lives of People with Learning Disabilities.

Inevitably I will only touch the surface of these vast areas of critical postcolonial and disability scholarship. In this paper I position Sylvia Wynter’s work as an entry point into a broader application of scholarship associated with postcolonial studies and the theory and activism of Black, Indigenous and People of Color (BIPOC). I am struck by the power of Katherine McKittrick’s (2015, p. 2) assertion that Wynter’s work exemplifies an approach where Blackness ‘is positioned not outside and entering into modernity but rather [as] the empirical-experiential-symbolic site through which modernity and all of its unmet promises are enabled and made plane’. Critical disability studies scholars have a similar hunch about Disability. The postcolonial centering of Blackness offers not only hope but also conceptual language and methodology to those of us who are interested in centering Disability and more specifically Learning Disability as a primary concern. I want to be clear from the outset that I am not conflating Disability with Blackness. There are fundamental differences in the ways in which Blackness and Disability have been historically rendered abject or desirable. However, I am drawn to the ways in which Blackness and Disability exist *together and in tension* with one another—creating opportunities for connections and also disconnections across embodied psychological and relational life.^4^ The centering of Blackness reminds me too of critiques of whiteness applied to my own field—critical disability studies scholarship—and the call for Black Disability Pedagogy in which the intersections of Disability and Blackness are brought together to formulate understandings with greater powers of critique (Dunhamn et al. 2015).

I also do not seek to replace Blackness with Learning Disability; rather to seek out moments of resonance. Take for example, McKittrick’s (2015, p. 2) suggestion that Wynter’s theorisation is aligned with ‘the colonised, impoverished, undesirable and lacking reason’, prioritising those who ‘currently inhabit the underside of the category of man-as-human’ to provide ‘a way to think about being human anew’. While People with Learning Disabilities are not explicitly mentioned by McKittrick, my sense is that they have too inhabit the underside of ‘the category of man-as-human’ (Ibid). Here is Wynter’s intersectional postcolonial gift: a theoretical and politicised language for honouring the lives of the disenfranchised. Postcolonial theory offers theoretical clarity to critical disability studies in affirming the lives of people so-labelled—to ‘adumbrate what is often hidden and ignored’ (Mignolo 2015, p. 115).

The interdisciplinary field of critical disability studies has matured into a scholarly, artistic and activist community that understands disability as a complex mix of biological, psychological, relational, social, historical, cultural, political, institutional, systemic, material, ecological and economic factors (Shakespeare 2013). Specific approaches have emerged, in various national contexts, that recognise disability’s minority status (North America), socio-economic foundation (United Kingdom), cultural location (Australia and North America), relational constitution (Nordic countries), bio-psycho-social character (supranational perspectives such as World Health Organisation and United Nations) and postcolonial influences (including Africa, Asia and South America) (see Mallett and Runswick-Cole 2014 and Goodley 2018 for discussion of these different models). Questions have been raised about the place of People with Learning Disabilities within disability studies (Chappell et al. 2001). Nevertheless, critical disability studies scholars have worked hard to depathologise, deindividualise and demedicalise dominant narratives that constitute disability in terms of human lack. Critical disability studies start with—but never ends with—disability. Contemplating Learning Disability offers up a number of exciting and hopefully liberating critical engagements with the human. Ndlovu (2021, p. 66) writes that postcolonial theory illuminates human emancipation through consciousness, increasing awareness of oppression and agency and fine-tuning us to reclaim humanness. These decolonising aspirations have much in common with the depathologising ambitions of critical disability studies.

In the *Souls of Black Folks* W.E.B. Du Bois [1903] (2012) wrote that the problem of the Twentieth Century is the problem of the colour-line. Sitting alongside postcolonial and disability theory, one could contemplate the Twenty-first Century as a continuation of the colour line and also what we might term the dis/ability complex (Goodley 2018). We know that disability is always racialised, gendered, classed, sexed and technologised. We also know that disability is the hidden referent to ability (and vice versa). Just as we have witnessed an exponential rise in the use of disability labels so we have witnessed a shoring up of the valuing, measurement and constitution of ability. Just as human difference is more likely to be understood in terms of disability vocabularies, so human value tends to be wrapped up in discourses of ability. The parallel constitution of disability and ability—the dis/ability complex (Goodley 2018)—is the formation of distinct though complementary processes of exclusion. When Disabled People are excluded from wider society then we can understand this as disablism (Thomas 2007). When able-bodied-and-mindedness is framed as a marker of human worth then we might understand this in terms of ableism (Wolbring 2008, 2012).^5^ As postcolonial scholarship disturbs the whiteness of social theory so critical disability studies disrupts the ableism within scholarship.

This paper is written by a non-disabled university professor who has spent the last thirty years working alongside People with Learning Disabilities in research (see Goodley 2020). While the vast majority of this empirical work adopts a co-production and participatory model, this article has not been co-authored with People with Learning Disabilities. I would describe my positionality as an ally, comrade and supporter of People with Learning Disabilities. I also share friendships and familial entanglements with people so-labelled. My personal and professional relationships have attuned me to some of the ways in which people so-labelled are invalidated by those around them: actualities that I will pick up on in this paper. I have also been lucky enough to be welcomed as an advisor to a self-advocacy group (Huddersfield People First) though this role ended almost two decades ago.

I do not aim to speak on behalf of People with Learning Disabilities but I am writing this paper *for* people so-labelled; to celebrate their activism whilst alerting us to the deeply ableist and disablist social contexts in which this human praxis emerges. I seek to offer critical theorisation that magnifies our understanding of their contributions to society. People so-labelled are routinely sidelined in the scholarship of transformative arenas such as critical pedagogy, critical and community psychology, feminist philosophy and social inclusion (Rapley 2004; Erevelles 2000; Goodley 2007; Carlson 2010; Richards et al. 2019; Clifford Simplican and Leader 2015). This is a missed opportunity. People with learning disabilities are being human in ways that challenge narrow forms of humanness that masquerade as the human per se. I call for the immediate recognition and celebration of *being human as praxis* enacted by many People with Learning Disabilities that offer generative versions of human being and being human.

## Method, study and analysis

The impetus for the writing of this paper came from an interdisciplinary research project funded by the Economic and Social Research Council entitled *Humanising the Healthcare Experiences of People with Learning Disabilities and/or Autism.* This project centres researchers with learning disabilities as research leaders; Barod (a workers co-operative) and three self-advocacy groups (Speakup Self-advocacy, Sunderland People First and Sheffield Voices). I reference some of the work of these four research organisations in my analysis; specifically, how they open up opportunities to ‘consciously and communally recreate ourselves; being human as praxis’ (Mynter and McKittrick 2015, p. 62). The politicisation of People with Learning Disabilities has historically been understood in terms of self-advocacy’s potential to organise, agitate and political struggle against the ideologies of ableism and disablism (Williams and Shoultz 1982). My analysis is framed by the research project’s problem (the dehumanisation of People with Learning Disabilities), specific predicament (the denigration of health and well-being of people so-labelled) and response (co-producing and identifying examples of humanising healthcare in consultation with researchers with learning disabilities). As a team we are committed to disseminating our findings to:

- People with Learning Disabilities, self-advocacy groups and their supporters and families;
- healthcare practitioners, service providers and policy makers;
- social science, humanities and clinical researchers

We use different formats including online presentations, Easy Read publications, illustrations, podcasts and journal articles. More details of our research and dissemination are provided on our project website: https://sites.google.com/sheffield.ac.uk/esrchumanisinghealthcare/home. This paper is an academic piece of writing. The analysis explore being human as praxis in (i) a culture of disposability; (ii) the midst of absolute otherness and (iii) the confines of a neoliberal-ableist society. For each of these three themes I start with a provocation, follow up with an exploration and end with a celebration (with the latter making reference to the activism of People with Learning Disabilities).

## Being human in a culture of disposability

Provocation No.1: That People with Learning Disabilities manage to exist in our communities is nothing short of astounding.

I make this provocation fully aware of the narrow confines assigned to the category of being human in our current times. According to Wynter (2003) this category is the Western bourgeois conception of the human—Man—that over-represents itself as if it were the human per se; as ‘the ostensibly only normal human’ (Wynter 2003, p. 265). In accounting for this over-representation as man-as-human, Wynter (2003) presents us with social and historical tracings of the emergence of what she terms Man1 (renaissance man, *homo politicus*) and Man2 (late Nineteenth Century liberal mono-humanist evolutionary man; *homo oeconomicus*). At the very moment that Columbus sets foot on^6^ Turtle Island in 1492 the concepts of man and human became one and the same (Mignolo 2015). Homo politicus is akin to Foucault’s sovereign self embodied by the rational political subject of the civilised European male. This is the coloniser with an already over-represented opinion of himself as normally human. By the late Nineteenth Century, the scientific subject—homo oeconomicus—is born; biologically and evolutionary developed, innately pristine and phylogenetically selected to survive and flourish. In collapsing man-human-normal, this constitution of humanness resonates with the phenomenon of the *normate* (Garland-Thomson 2012; Titchkosky 2022). The figure of the normate dominates humanist conceptions of man/human in modern Western European secular societies—personified by Leonardo da Vinci’s image of Vitruvian Man—and detectable in the most contemporary representations of popular culture.^7^ Mpofu and Steyn (2021, p. 1) assert that the ‘grand construction of *the human* of Euro-modernity was founded on unhappy circumstances and for tragic purposes. That which was categorised as non-human became things, reduced to resources, usable and disposable by the unapologetic humans*’*.

One unhappy circumstance, following Razack (2016), relates to disposability: the transformation of some human beings into waste. Disposability is but one by-product of dehumanization—the movement from human subject to object—the reduction of a human population to shapeless matter that is to be discarded. Undergirding the constitution of use/waste is a ‘Western rationality and enlightenment’ that ‘became complicit in the irrational and dark oppression of black people, whose humanity was carefully doubted and opportunistically dismissed’ (Mpofu and Steyn (2021, p. 11). The dismissal—laying waste to certain types of humanity—is a consequence that migrates between and across Black and Disabled People.^8^ The Covid-19 pandemic brutally exposed the disposability of People with Learning Disabilities. Blanket ‘Do Not Resuscitate’ orders, controversial measures of Clinical Fraility and the care home crisis invalidated the lives of many more; increasing levels of psychological distress, social isolation, a lack of social care, workplace discrimination, food poverty, and unequal access to health care’ (Shakespeare et al. 2022). By Autumn 2020, People with Learning Disabilities and/or autism were six times more likely to die from Covid-19 than the rest of the population (Public Health England 2020). By 2021 Covid-19 was the leading cause of death for people with a learning disability (White et al. 2020). The pandemic gave life to the lie that disability equates with disposability.

Wynter's being human as praxis emphasises Black humanity as livingness; as direct counter-distinction to any notion that Blackness equates with disposability (Wynter and McKittrick 2015). Similar counterpoints to the implicit cultural narrative that Disabled lives are disposable lives are provided in the work of The Rightfullives Exhibition:

HUMAN RIGHTS, LEARNING DISABILITY AND AUTISM

A COMMUNITY OF PERSPECTIVES

Welcome to the Rightfullives online Exhibition. It’s an exhibition that explores the theme of Human Rights and people with autism and/or learning disabilities. The idea for the exhibition came about through a conversation about how the legal framework of the Human Rights Act seems to barely touch the lives of People with Learning Disabilities. Since then we have only been able to find three published successful court judgements where the HRA has been applied to learning Disabled People. So in May 2018, we put out a call to arms. We asked for contributions from anyone interested in human rights for learning Disabled People. The responses, from an incredibly diverse group of people, have been phenomenal.

This exhibition is the result of that call to arms. We hope you enjoy it.

The exhibits are full of joy and laughter, anguish, pain and occasionally strong language. They will make you smile, cry and rage. We feel that we must warn you that some people may find some of the exhibits distressing.

https://rightfullives.net

Similarly, the reclaiming of rightful lives is exemplified by the community work of Sheffield Voices in their generative practices that brought together People with Learning Disabilities to share and value lives worthy of telling and storying:

*Magic Pen Group*

The Magic Pen writing group is a collaboration between Disability Sheffield and Burton Street Foundation.

It is a friendly online group for anyone with a learning disability who loves stories and having fun with words. Every week on Zoom, we talk about different topics, have fun together, share our news.

We write poems and stories together, and there is always time to share your creative writing too.

There's no need for you to write anything down if you don't want to - just come along and join our chat! Please fill in the form on the bottom of page or press the link *Contact Form*

Click on the stories to see our work.

You can check out the *Magic Pen Videos* made by the group too.

https://www.sheffieldvoices.org.uk/magic-pen

The being human as praxis of Sheffield Voices and The Rightfullives Exhibition captures what Ryan (2020) beautifully describes as pockets of brilliance—those moments when People with Learning Disabilities are valued on their own terms by those around them. The key here is to move pockets of brilliance to enabling communities; a form of campaigning, support and recognition that might be understood as examples of a ‘disability commons’ (Runswick-Cole and Goodley 2017; Runswick-Cole and Ryan 2019). A disability commons is antithetical to the dominant discourse of disability as disposable. While it is incredible that People with Learning Disabilities still populate our post-pandemic communities; this existence is precarious. The search for moments of being human as praxis continue—and need to continue—because of the threat of being cast as human waste.

## Being human in the midst of absolute otherness

Provocation No.2: People with Learning Disabilities continue to survive in times when their very existence has been put into question.

One history for People with Learning Disabilities is that marked by stigmatisation, marginalisation and systemic abuse (Edgerton 1967; Bogdan and Taylor 1976; Langness and Levine 1986). The lack of moral authority and personhood ascribed to People with Learning Disabilities can be directly correlated with their segregation and institutionalisation (Vehmas and Mietola 2021). This critical disability studies take on the constitution of People with Learning Disabilities as peripheral outsiders resonates with Wynter’s conception of the Wholly Human Other status ascribed to those that do not fit ‘the Western bourgeois liberal mono-humanist conception of the human’ (Wynter and McKittrick 2015, p. 45). When something is rendered Absolute Other it is dismembered and thingified. In writing of colonialism Ndlovu-Gatsheni and Ndlovu (2022, pp. 29–30) argue:

the concept of dismemberment speaks to how those who were ‘othered’ as black people were pushed out of the human family, and underscores the very denial of their humanity (their thingification) … Dismemberment is part of the unfolding and expansion of Euro-North American-centric modernity, which in practice involved submission of the modern world to European memory … Exploration, surveying, ‘discovering’, mapping, conquest, colonisation, naming, dispossession and claims of ownership of everything in the modern world formed the core of dismemberment

Postcolonial theory grapples with how the Absolute Other is constituted through a coloniality of being whilst also offering responses to reclaim humanness of the Absolute Other (Ndlovu 2021, p. 68). This work resonates with the human being as praxis of People with Learning Disabilities. A stark reminder of one’s absolute otherness is found in death. In October 2022 the *Learning from lives and deaths—people with a learning disability and autistic people report* (LeDeR) found that less than 2 of every 10 people that die in the general population will be younger than 65 while 6 in 10 of people with a learning disability die are under 65 (White et al. 2022). 49% of deaths of People with Learning Disabilities were rated as ‘avoidable’ compared with 22% of the general population. 8% of these avoidable deaths were linked to cancer, 14% to hypertension, 17% to diabetes and 17% to respiratory conditions (Ibid). An avoidable death or preventable mortality refers to causes of death that can be mainly avoided through effective public health and primary prevention interventions (ONS 2021). As the disability studies researcher Porter (2020) has argued, Disabled People are subjected to forms of governance and policy making that in effect control how some people can live while others can die. In Mbembe’s (2003, p. 11) postcolonial work on necropolitics, they argue that ‘the ultimate expression of sovereignty resides, to a large degree, in the power and the capacity to dictate who may live and who must die’. While Porter (2020) suggests that Disabled People are part of the metaphorically ‘living dead’, Covid-19 moved too many towards a literal death.

In the face of this existential crisis, self-advocacy groups rallied, self-organised and delivered a number of humanising responses. Here are two examples:

*Rate my healthcheck*

People with Learning Disabilities often have difficulty in recognising illness, communicating their needs and using health services.

Regular health checks for People with Learning Disabilities often uncover treatable health conditions. Most of these are simple to treat and make the person feel better.

All people with a learning disability are entitled to annual health checks. Sunderland Action for Health want to know what people think about their experiences of having a health check.

GP’s will be asking people to fill in a very brief questionnaire or answer an online questionnaire.

http://sunderlandpeoplefirst.com/rate-my-healthcheck/

*Peaceful Minds*

Peaceful Minds is a lottery funded and a co-produced project between Speakup Self Advocacy and Rotherham Advocacy Partnerships. We are working together to support People with Learning Disabilities and autistic people who also have mental health conditions and anxieties. The project offers crisis intervention and support. The peer supporters and inclusion workers support a person to look at coping strategies, self-soothing ideas and solutions to give the individual affected a better quality of life. The team also works with people to make sure that they are receiving the correct Benefits which they need to live a good life and stay out of crisis.

https://www.speakup.org.uk/peacefulminds

These organisations grappled with the necropolitical situation of People with Learning Disabilities; a post-pandemic actuality in which the very existence of People with Learning Disabilities has been brought into question. Sunderland People First and Speakup not only offer practical solutions to promoting health checks and offering crisis intervention and support: they reclaim the lives of People with Learning Disabilities. Being Human as praxis is normalised in these self-advocacy groups; rehumanising People with Learning Disabilities in times where their very being as humans is threatened.

## Being human in the confines of a neoliberal-ableist society

Provocation No. 3: In a rapidly developing, competitive and deeply individualistic capitalist society, People with Learning Disabilities somehow still manage to live, love and labour.

Against the backdrop of disposability and absolute otherness, People with Learning Disabilities have to grapple with an especially demanding social, economic and political context. As we enter a cost-of-living crisis we know that some citizens will fare better than others. One way of characterising our contemporary times is to understand them as being under-girded by an ideology of neoliberal-ableism; a dominant cultural imaginary and social symbolic (exacerbated by the pandemic) that values the mobile, self-sufficient, responsible, accountable and flexible normative citizen (Goodley 2014). Neoliberal-ableism is a cultural construction of critical disability studies but one that resonates with postcolonial critiques of colonial capitalism where ‘categorisation and labelling of humanity in terms of the normative standards set by dominant powers constructs certain humans as the others’ (Ndlovu 2021, p. 72). Neoliberal-ableism sits readily alongside forms of colonial humanism ‘that makes the will to power, the desire to climb up above others, critical for progress, at the expense of the will to co-exist with others in conditions of peace, love and justice page’ (Zondi 2022, p. 227). This humanism ‘is a form of humanism that, while masquerading as recognition of the humanity of many, permits the violation of the humanity of others’ (Zondi 2022, p. 228). As Sithole (2022, p. 129) writes ‘If the human is a given, then the human exists in the world. In a sense, the human is inseparable from humanity; for there to be humanity there must be life. However, the mutual standing of the human and humanity has allowed an ontologically absurd separation, one that suggests the human can exist without humanity’. This suggestion that the human can exist without humanity demonstrates the problems with only thinking of the human as a noun. Instead, we need to consider human being as enactment or praxis.

Following Wytner, McKittrick (2015, p. 7) demands that we engage in the difficult labour of thinking the world anew. Cognition and action, of course, come together especially when one considers the sheer extent to which People with Learning Disabilities have created ‘new stories of being human that challenge the profitable brutalities’ of colonial capitalism that profit from margination (McKittrick 2015, p. 7). As Mpofu and Steyn (2021, p. 16) argue, ‘a need has arisen for humanity to depart from the European and Western colonial example of power and knowledge, to champion other human directions in pursuit of liberated futures. Liberation from oppression, therefore, demands the rehumanisation of both the oppressor and the oppressed’. A liberatory discourse is offered by Barod—a workers cooperative—that emphasises the labour and contributions of People with Learning Disabilities:

There is a point where you just have to go for it. You have to have faith in yourself and faith in those around you.

That's how a number of People with Learning Disabilities have described the point, where they went for their first paid job, or where they crossed the 16 hours a week threshold.

It's so easy to stay where you are, it's quite exhausting fighting with the benefits system with fears about losing benefits, because of 'fitness to work' assessments that make no sense, or sorting out what support you're entitled to from social services. So why would you jeopardize all that by getting a job that pays real money?

Well, that's what Alan Armstrong did, 8 years ago.

With four friends he set up Barod as a workers cooperative and started a 16 hours a week job. Alan had a job coach for a while, but that didn't work out well. So, one of the other workers provided support and Alan learnt how to make dictation software work for him and got his head round Photosymbols, photos, that can be used to support written words to make the meanings clearer.

Barod started by renting a small room as an office in the Carmarthenshire, at 'People First building'. They used the training room for group work and skyped with workers who worked from home. They have moved to a bigger room to this day, but still in the friendly surroundings of 'Carmarthenshire People First'.

And what about the four friends that made up the five letters of Barod? Well, one was told by his job coach at the JobCentrePlus, that being in a company and a company director was not for him, so then there were four.

After a year, or so, one of the other worker/directors decided that he didn't want to stay in Barod and left. Then there were three, still standing, reformed the workers cooperative into a community cooperative. So they could have members and allies involved, and workers didn't have to be directors.

This meant that Barod could grow, and benefit from different skills and experiences. Barod now employs 8 people, has 5 directors and 20 members.

Barod has always had fun at its heart! From posting Jaffa cakes to people who helped out on social media, wearing hats in skype meetings so everyone knew what role you are doing, to crazy activities in workshops involving hoops and sitting important people on the floor.

Barod started by making information from public bodies accessible to People with Learning Disabilities. It still does that, setting up Planet Easy Read, so more people can get involved and earn some money.

This led to thinking about how government consultations work and developing new methods to make them effective. And then, there was the research.

Research into employment and being a business person with a learning disability, research into self advocacy and what makes a good self advocacy project!

Barod has also done some Big Thinking, drawing people together to get their ideas into action, around co-production, health passports and getting involved in hack days.

Barod has also led projects to develop new apps and services that enable People with Learning Disabilities to lead their lives the way they want.

In 2019, after 6 years of trading, Barod passed the half million pound turnover milestone, and has restructured to concentrate on developing new products to sell to private businesses and to break into the world of academic research.

Sadly, Alan died recently, so his active part in Barod has ended. His leap of faith led him to move on from seeing himself as a self advocate to a worker and director in a business and then further as a researcher. He was always ready—barod in Welsh—for the next challenge and we will keep that spirit going, as we go forward to the next challenge

https://www.barod.org/hello-world/

Alan’s story brings us back to the celebration of human being as praxis enacted by People with Learning Disabilities. That he and his comrades were able to do this in the context of neoliberal-ableism makes their achievements even more remarkable. Alan’s story captures Mpofu and Steyn (2021, p. 16) demand ‘to champion other human directions in pursuit of liberated futures. Liberation from oppression, therefore, demands the rehumanisation of both the oppressor and the oppressed’. His narrative moves him from a position of deficit as he discusses having faith in his own humanity: a humanity emboldened by his connections with others in the Barod organisation. A humanity that is ‘ready’—‘barod’ in Welsh—for the next challenge lived in concert with his comrades.

## Discussion: towards decolonisation and depathologisation

I have sought to demonstrate how the human praxis of People with Learning Disabilities is sophisticated and generative; enacted in a deeply disablist and ableist world. Their praxis demands our recognition; propelling us all to think again about how we theorise, research and understand what it means to be human. I find it helpful here to draw on a postcolonial project—specifically Zondi’s (2022) five decolonising practices—to consider the reach and potential of being human as praxis of People with Learning Disabilities.

First, is the importance of *embracing our relational selves*. Zondi (2022, p. 237) argues that we ‘embrace in earnest and in practice the ways of being long provided for in indigenous paradigms of being, such as ubuntu’. The importance of relationality has been picked up on and extended in the work of critical disability scholars (Reindal 1999; Shakespeare 2000). As with indigenous communities, disability communities have always historically engaged with relational interdependencies that challenge the individualising tendencies of neoliberal-ableism. This work—some of it described in the above analysis—contributes to a broader project of depathologisation and decolonisation (see also Goodley and Ktenidis, 2023).

Second, is the requirement for a *mutual recognition of the humanity of others *(see also Cornell and Van Marle 2015). This entails ‘being and doing human as a process of restoring, enriching and reinforcing the humanity of others, through our speech, the ways we relate to others, and the design of human systems’ (Zondi 2022, p. 238). The aims, objectives, language and sentiments of Barod, Speakup, Sunderland People First and Sheffield Voices described above capture in detail some of the ways in which People with Learning Disabilities engage in human being as praxis to offer design, support and mutuality.

Third, is an emphasis on *communalism* ‘understood principally as a way of living, of co-existing and working with others. It requires conscious efforts to function in ways that build communities and communal practices instead of perpetuating esoteric individualism that breaks human bonds’ (Zondi 2022, p. 239). My brief insight into the work of People with Learning Disabilities captures a particular kind of communalism that contrasts markedly with disposability, absolute otherness and neoliberal-ableist exceptionalism.

The fourth practice involves* endeavouring to ‘achieve human excellence with humaneness’* (Zondi 2022, p. 239). The storytelling of Sheffield Voices and joyful aspirations of the The Rightful Lives Exhibition seek to reaffirm the human and the human in the lives of People with Learning Disabilities. We are reminded that societal responses to People with Learning Disabilities are too often the direct opposite of the very idea of humane; and therefore require our urgent attention.

Fifth, the task of humans always ‘goes’ (Zondi 2022, p. 239). All of the organisations cited in this paper engage in very practical work with People with Learning Disabilities. However, we should not confuse the practical with the atheoretical. Instead, their critical interventions push me to consider how we might think through and with decolonisation and depathologisation: a broad project of challenging ableism and disablism in knowledge production. Again, I have no desire to conflate nor collapse these processes of decolonisation and depathologisation; rather to consider how the two processes might support one another to create a new humanism that is anti-bourgeois, anti-colonial, anti-imperial, anti-global (Zondi 2022, p. 232) and, may I add, anti-ableist and anti-disablist. In seeking our new dialogues about new humanisms we need ‘to rediscover how to enter into genuine dialogues such that the ways of living of different human groups are mutually enriched, without one way of living disappearing in the face of another’ (Zondi 2022, p. 233).

## Conclusion: on writing *for* rather than *with*

Scholar-activists such as Simone Aspis who identifies as a researcher with learning disabilities has expressed grave concerns with the very idea of others speaking for and on behalf of people without learning disabilities (Aspis 2022). While I do not seek to speak for People with Learning Disabilities, my voice dominates this paper. I am very much aware of the critiques of powerful people speaking on behalf of less powerful others. I acknowledge the paradox inherent in an enterprise (the writing of an academic paper) owned solely by an established academic. I am aware too of the debates that have ensued in relation to the ethics of others writing for and on behalf of People with Learning Disabilities. I will not rehearse these arguments here but instead encourage readers to follow up on the work of Eva Feder Kittay, Katherine Runswick-Cole and Sara Ryan (Kittay 1999; Ryan and Runswick-Cole 2008; Unwin 2022). These parent-scholar-activist authors hold familial positions that are much closer to the lives and lifeworlds of People with Learning Disabilities than my own. And yet they still face the challenge of justifying their own perspectives in speaking with and for their children. Postcolonial and critical disability studies scholarship trouble questions of positionality and voice in research: demanding us to consider who is speaking for, with and alongside one another.

Social theorists, researchers and academics have long failed in their theoretical endeavours because they have either implicitly or explicitly excluded People with Learning Disabilities. The lives, aspirations and contributions of People with Learning Disabilities are often deemed unworthy (Taylor 2013). And unworthy lives often become non-theorisable lives; excluded from theory and absent in the curricula of disciplines across the human, social and natural sciences. We need to sit with and respond to the being human as praxis of People with Learning Disabilities; otherwise we will fail to attend to their generative contributions. Researchers, scholars and activists in the field of critical disability studies have epistemological, ontological, methodological, moral and political duties to take seriously the human praxis of People with Learning Disabilities. And in order to attend to this work then one requires the requisite conceptual tools and theoretical language for making sense of this labour.

My hope is that this paper is one small contribution to this politics of recognition. But this is only one part of the picture and we must also attend to the theoretical contributions authored by People with Learning Disabilities. This work is further evidence, if it was ever needed, that People with Learning Disabilities are theorists, researchers and analysts in their own right. There is now a burgeoning literature in the human and social sciences documenting the opportunities afforded by democratising the research enterprise and bringing in People with Learning Disabilities as depathologising researchers in their own right (for examples see Kiernan 1999; McClimens 2007; Chapman et al 2014; Nind et al 2016; Murray 2019; Liddiard et al 2019; Clifton and Chapman 2020). In sitting with theorisations of the human and learning disability in this paper, we are reminded of the need to position People with Learning Disabilities not simply as research partners, the co-producers of research inquiry or as equal participants in methodological endeavour. We need also to respond to and recognise them as theorists in their own right. Sitting with their being human as praxis informs this recognition. In working with some of Wynter’s ideas, I am reminded of McKittrick’s (2015, p. 3) call for a new science—one that will ‘always be cast by the shadow of the Man-as-human but demands a project outside of our present order of knowledge’. In contemplating the contributions of People with Learning Disabilities, I ask you: do you understand their being human as praxis?

## Footnotes

1 The term learning disabilities is the one used in the UK with other labels being used in different countries ranging from development disabilities, intellectual disabilities and cognitive impairments. I will refrain from offering a mainstream administrative definition of learning disabilities—which would normally refer to issues of competence, intelligence and maladaptive functioning—in response to the wider aims of this paper to centralise people so-labelled in broader and more affirmative discussions of humanity.

2 I have deliberately capitalised People with Learning Disabilities, Black and Disabled People to recognise their centrality to this paper and the scholarship introduced in this paper.

3 Described by Sylvia Wynter in her conversations with Katherine McKittrick–Wynter and McKittrick (2015). In. McKittrick (Ed). (2015).

4 I am indebted to conversations with Tanya Titchkosky and Rod Michalko for furthering my understandings of some of these dis/connections.

5 While it is possible to distinguish between ableism and disablism it is important to acknowledge that these ideologies are intertwined and mutually reinforcing. My thanks to an anonymous reviewer for urging me to clarify this point.

6 One could sit and ponder the assumed physical mobility and normativity here associated with ‘setting foot’—the ableist assumption embodied in the coloniser effortlessly walking from boat to land ‘discovered’. See Oliver’s (1993) classic challenge to the presumption of walkers and walking.

7 See the especially uber-white-humanist depictions of fathers and sons in the Patek-Philippe watch advertisements for a particular horrific example e.g.https://www.forbes.com/sites/robertanaas/2016/12/09/patek-philippe-celebrates-20-years-of-its-iconic-advertising-campaign-you-never-actually-own-a-patek-philippe/?sh=17a4ea19475b.

8 It is worth acknowledging that numerous social theorists and public intellectuals have attended to these matters of human life and death; unearthing the many reasons why some human beings are afforded positions of privilege while others are rendered wasted, precarious, bare, dead, disposable or debilitated (Giroux 2006; Mbembe 2003; Agamben 1998; Bauman 2004; Butler 2004, Puar 2009).
